# Animal Abuse Reporting and the Ethical Role of Veterinarians: A Comparative Review of Practices in South Korea, Canada, and the United States

**DOI:** 10.3390/ani15233408

**Published:** 2025-11-26

**Authors:** Gina S. Rhee, Rahyeon Ahn

**Affiliations:** 1 The Catholic College, The Catholic University of Korea, Bucheon 14662, Gyeonggi-do, Republic of Korea; 2 Department of Small Animal Surgery and Medicine, Ontario Veterinary College Health Sciences Centre, Guelph, ON N1G 2W1, Canada; rahyeonahn@gmail.com

**Keywords:** animal abuse reporting, veterinary ethics, mandatory reporting, South Korea, Canada, United States, comparative analysis, animal welfare

## Abstract

Animal abuse reporting represents a crucial ethical responsibility for veterinarians globally, with cultural, legal, and societal factors significantly impacting practices. This comparative narrative review investigates veterinary reporting practices across South Korea, Canada, and the United States. A narrative review was conducted using systematic searches across PubMed, Google Scholar, Web of Science, and Scopus databases, employing jurisdiction-specific terms. Additional searches encompassed state-specific legal databases, professional licensing board records, and disciplinary action reports. To investigate reporting systems, enforcement mechanisms, and outcomes, legal documents, policy analyses, and empirical studies from 2010 to 2025 were analyzed. Findings revealed stark contrasts between voluntary and mandatory systems. South Korea’s voluntary framework caused significant underreporting. Canada’s provincial mandatory frameworks exhibited significantly higher levels of compliance, as a result of the strength of their statutory enforcement mechanisms. The United States presented a mixed landscape, wherein approximately 24 states mandated reporting with license revocation penalties, whereas others maintained voluntary systems. Cultural factors, professional autonomy concerns, and enforcement mechanisms shaped reporting behaviors and animal welfare outcomes. South Korea requires legislative changes implementing graduated mandatory reporting systems with legal protections and cultural adaptation strategies. Benchmarking against Canadian and American models demonstrates that mandatory reporting with appropriate enforcement mechanisms shows potential to enhance animal welfare protection through increased reporting rates and systematic intervention pathways, though direct causal evidence linking reporting to measurable welfare outcomes remains limited.

## Simple Summary

Veterinarians play an essential role in identifying and reporting animal abuse. However, reporting practices vary widely across countries. This study compares the systems in South Korea, Canada, and the United States to understand how laws, cultural values, and professional expectations shape veterinarians’ actions when they encounter suspected abuse. Canada and many U.S. states require veterinarians to report abuse and provide legal protections for doing so. These systems typically lead to higher reporting rates and greater support for animal welfare. In contrast, South Korea currently uses a voluntary reporting approach, resulting in lower reporting rates due to social pressures, limited legal protection, and cultural barriers. Our findings indicate that adopting a gradual mandatory reporting system in South Korea—supported by legal safeguards, professional training, and public education—could improve animal protection. Nonetheless, further research is needed to determine how increased reporting directly affects long-term animal welfare outcomes.

## 1. Introduction

Animal abuse signifies a pressing global issue with profound consequences for animal welfare and human society, establishing compelling associations with interpersonal violence and broader public safety issues [1]. The veterinary profession occupies a unique position in detecting and preventing animal abuse as veterinarians frequently function as the initial interface for animals experiencing neglect, physical trauma, or psychological distress [2]. This professional role fosters multifaceted ethical obligations that significantly vary across jurisdictions, influenced by interactions between legal mandates, cultural norms, professional training, and enforcement mechanisms.

The theoretical foundations for understanding veterinary obligations in animal abuse reporting draw from varying philosophical traditions. Utilitarian approaches highlight the minimization of suffering, likely supporting reporting systems that maximize overall animal welfare outcomes. Rights-based theories recognize animals as subjects of moral consideration, generating strong obligations for veterinarians to report suspected abuse. Virtue ethics approaches focus on the character and professional integrity of veterinarians, underscoring compassion, courage, and advocacy as essential professional virtues. The “link” theory, extensively documented in criminological and veterinary literature, establishes compelling associations between animal abuse and interpersonal violence, suggesting that veterinarians’ role in identifying and reporting abuse extends beyond animal welfare to encompass broader public safety concerns [3]. This association has influenced legislative developments across jurisdictions, with varying approaches to mandatory reporting requirements and professional obligations [4].

Veterinary reporting practices currently exhibit substantial international variations. Approximately 24 states in the United States have implemented mandatory reporting requirements for veterinarians, frequently with professional disciplinary consequences, including license suspension or revocation for noncompliance [5]. Canada operates comprehensive provincial mandatory reporting systems with reputable legal protections and institutional support [2]. In contrast, South Korea maintains a voluntary reporting framework relying on individual veterinarian discretion and professional ethical guidelines, influenced by conventional cultural values and contemporary social dynamics. This comparative analysis aimed to investigate these three distinct approaches to veterinary reporting obligations, exploring how cultural values, legal frameworks, and enforcement mechanisms impact professional practice and animal welfare outcomes.

## 2. Methods

To investigate veterinary animal abuse reporting practices across South Korea, Canada, and the United States, a comprehensive narrative review methodology was employed. To ensure comprehensive coverage of relevant literature and legal documentation, systematic literature searches were conducted across various databases, including PubMed, Scopus, Web of Science, Google Scholar, and specialized Korean academic databases (KCI and DBPIA), as well as legal databases, including Westlaw, LexisNexis, and state-specific legislative archives. Search terms were developed through iterative refinement and comprised combinations of keywords, such as “animal abuse reporting,” “veterinary ethics,” “mandatory reporting,” “South Korea animal welfare,” “Canada veterinary legislation,” “United States veterinary reporting laws,” “animal cruelty detection,” “cross-cultural veterinary practices,” “license revocation,” “disciplinary action,” and “professional misconduct.” The search strategy employed English and Korean language terms, with Korean literature accessed to ensure comprehensive coverage of domestic research and policy documents.

Peer-reviewed articles, legal analysis, policy documents, government reports, and case studies directly addressing animal abuse reporting practices in veterinary contexts were included. Studies were required to specifically focus on veterinary involvement in abuse detection or reporting, include ethical considerations, and provide relevant data on practices in the target jurisdictions. Legal documents comprised significant legislation, regulatory guidelines, and judicial decisions impacting veterinary reporting obligations.

To focus on contemporary practices while capturing the evolution of animal welfare legislation across jurisdictions, the search timeframe was limited to 2010–2025. Publications were systematically screened for relevance, with 69 sources ultimately selected following detailed review procedures, including 25 legal documents, 29 peer-reviewed articles, 10 government reports, and 5 professional organization publications. Data extraction focused on legal frameworks, enforcement mechanisms, compliance rates, cultural factors, and professional outcomes across the three jurisdictions. The distribution of these documents across jurisdictions is summarized in Table 1.

## 3. Comparative Analysis of Reporting Systems

### 3.1. South Korea: Voluntary Reporting Framework

South Korea’s animal protection legal framework relies on a voluntary reporting system established under the Animal Protection Act, originally enacted in 1991 with major amendments in 2017 and 2021 [6]. Recent legislative developments have strengthened penalties for animal abuse while maintaining the voluntary nature of veterinary reporting, reflecting ongoing tensions between conventional cultural values and evolving animal welfare consciousness. Empirical research reveals considerable gaps between witness rates and reporting behaviors among Korean veterinarians. Joo et al. [7] reported that 86.5% of surveyed veterinarians had encountered suspected cases of animal abuse in their practice, representing one of the highest witness rates documented worldwide. However, only 48.6% of these veterinarians expressed willingness to report such cases to authorities (“definitely would,” 15%; “probably would,” 33.6%), underscoring significant underreporting within the voluntary system.

Han [8], in cross-cultural psychology research, explained that the notion of “face” in Confucian societies generates social pressure that discourages reporting misconduct. The study revealed that fears of social conflict and damaging relationships frequently override professional duties in East Asian settings [8]. Additionally, Cho and Ha [9] pointed to systemic issues in Korea’s animal welfare policies, noting that there is a widespread lack of emergency awareness concerning animal welfare. Their findings indicated that this “emergency unawareness” affects government bodies, veterinary practitioners, and the general public, resulting in weak infrastructure for protecting animals [9].

This underreporting occurs within a context of rapid growth in pet ownership, with data from the Korea Ministry of Agriculture, Food, and Rural Affairs indicating that 25.4% of Korean households owned pets in 2022, representing 6.02 million households and a 65% increase from 3.64 million households in 2012 [10]. The cultural context significantly influences reporting practices, with Korean veterinarians navigating complex social dynamics that prioritize relationship harmony and face-saving behaviors, frequently conflicting with formal reporting obligations.

### 3.2. Canada: Provincial Mandatory Reporting Systems

Canada operates comprehensive mandatory reporting systems that primarily function at the provincial level, with significant consistency in reporting requirements and enforcement mechanisms across jurisdictions. In Canada, nine provinces (excluding Alberta) have statutory requirements that compel veterinarians to report animal mistreatment to official enforcement arms of provincial justice systems [11]. Ontario’s framework represents the most comprehensive strategy, necessitating veterinarians to report suspected animal abuse and provide robust legal protections against civil liability. Ontario operates mandatory reporting and good-faith immunity under the Provincial Animal Welfare Services Act (2019) and College of Veterinarians of Ontario standards using a “reasonable grounds” criterion [12].

The Canadian system offers comprehensive legal protections for veterinarians who report cases, including immunity from civil liability, protection against professional discipline when reporting in good faith, and institutional support for veterinarians participating in legal proceedings. Provincial veterinary colleges offer ongoing ethical guidelines, training programs, and institutional support for reporting obligations, fostering systematic approaches to professional education and compliance monitoring [2].

### 3.3. United States: State-by-State Variation with Enforcement Mechanisms

**Figure 1:**
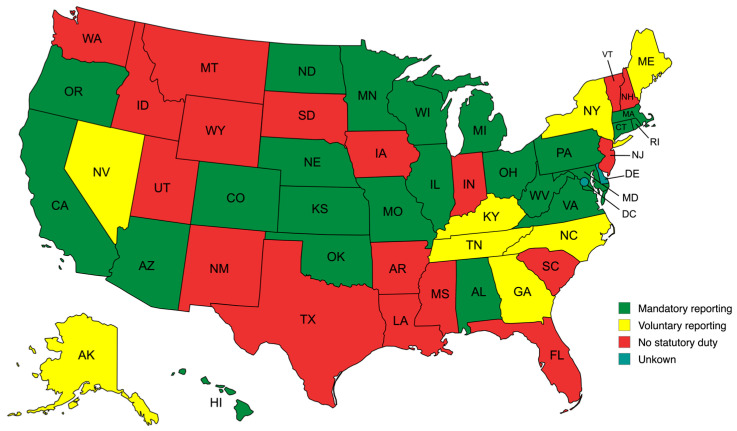
States-by-state variation animal abuse reporting laws in the United States.

The United States has a multifaceted approach to veterinary reporting requirements, with an evolving number of mandatory reporting states. Recent analyses have suggested approximately 24 states implement mandatory reporting duties for licensed veterinarians [5], indicating an increase from the 19 states documented in 2020 [13,14]. A summary map of the United States with and without statutory mandatory-reporting requirements for veterinarians is presented in Figure 1.

California’s framework demonstrates comprehensive mandatory reporting requirements. California Business and Professions Codes §§4830.5–4830.7 oblige licensed veterinarians to promptly report suspected animal fighting and abuse to law enforcement, with failure to comply constituting unprofessional conduct subject to disciplinary actions [15]. The California Veterinary Medical Board has documented disciplinary guidelines specifying maximum penalties of license revocation and $5000 fines for violations of reporting duties [16]. Other states, including Kansas, Oklahoma, and North Dakota, where administrative codes specifically define failure to report as “unprofessional conduct,” constituting grounds for disciplinary actions, including license suspension or revocation, employ similar mandatory frameworks.

## 4. Mandatory Reporting States with License Revocation Penalties

States with mandatory reporting requirements frequently implement graduated disciplinary measures for noncompliance. California Business and Professions Codes §§4830.5–4830.7 require licensed veterinarians to promptly report suspected animal fighting and abuse to law enforcement, with failure to comply constituting unprofessional conduct subject to disciplinary actions, including license suspension or revocation [15]. The disciplinary guidelines of the California Veterinary Medical Board stipulate maximum penalties of license revocation and $5000 fines for violations of reporting duties [16].

Kansas Administrative Regulations §70-8-1 define failure to report cruel or inhumane treatment as “unprofessional conduct,” constituting grounds for disciplinary actions [17]. Veterinarians in Kansas should report cases where they have “direct knowledge” of cruel treatment, with violations potentially leading to license suspension, revocation, or refusal to renew. The Oklahoma Administrative Code 775:10-5-30(8) requires veterinarians to report inhumane treatment, with failure to report constituting grounds for disciplinary actions [18]. The Oklahoma Veterinary Medical Examining Board has authority to impose penalties ranging from reprimands to license revocation for noncompliance.

The Massachusetts General Laws ch. 112 §58B mandates reporting of suspected cruelty, with noncompliance constituting grounds for disciplinary actions under the Board of Registration in Veterinary Medicine [19]. The Minnesota Statutes §346.37 subd. 6 necessitates mandatory reporting for known or suspected cases of abuse, cruelty, or neglect, with noncompliance establishing grounds for disciplinary actions under professional conduct regulations [20]. It indicates that states with clear disciplinary frameworks demonstrate higher compliance rates and more systematic approaches to animal welfare protection.

Cultural factors play a significant role in shaping attitudes toward animal welfare reporting. Urbanized populations—characterized by higher levels of education, exposure to companion animals, and social awareness—typically demonstrate stronger empathy toward animals and greater concern for their welfare [21,22]. In contrast, in regions with deep-rooted agricultural or ranching traditions, there is often a cultural preference for professional autonomy and self-regulation over external oversight. These cultural distinctions can meaningfully influence how animal mistreatment is perceived, prioritized, and acted upon within different communities.

Institutional and professional factors further complicate the effectiveness of mandatory reporting. Even in jurisdictions where legal mandates are in place, actual reporting behavior is heavily shaped by the norms and values upheld within professional veterinary practice. The presence (or absence) of immunity provisions for veterinarians can significantly influence whether professionals feel secure enough to report suspected abuse without fear of legal or professional repercussions [4]. These institutional dynamics highlight the need for well-structured support systems, clear guidelines, and robust legal safeguards to ensure compliance and protect both practitioners and the animals they serve.

## 5. Cultural and Professional Factors Influencing Reporting Practices

### 5.1. Cultural Dimensions and Professional Decision-Making

Comparative analysis reveals fundamental cultural differences impacting veterinary reporting practices across jurisdictions. South Korea’s cultural context, influenced by Confucian values highlighting social harmony, hierarchical relationships, and collective well-being, fosters professional environments where veterinarians frequently prioritize maintaining relationships over formal reporting mechanisms [23]. Conventional Korean perspectives on animals, historically viewing them primarily through utilitarian lenses, continue to affect contemporary veterinary practices despite rapid changes in pet ownership and animal welfare consciousness.

In South Korea, Han [8] has identified specific cultural barriers to animal welfare reporting, including concerns about social disruption, fear of economic retaliation from clients, and conventional hierarchical systems that discourage challenging authority figures. The digital dimension of Korean culture fosters unique reporting barriers absent in other jurisdictions, with veterinarians conveying concerns about online reputation attacks and social media harassment after reporting abuse.

In contrast, Canadian and American cultures exhibit greater historical integration of animal protection values into professional practice frameworks. The gradual development of animal protection legislation in these jurisdictions, beginning with early 20th-century anti-cruelty laws, developed cultural foundations supporting contemporary mandatory reporting systems [4]. In these jurisdictions, professional cultures generally accept animal welfare as a fundamental veterinary obligation that may supersede individual client relationships in cases of suspected abuse. To facilitate cross-jurisdictional comparison, Table 2 provides a simplified overview summarizing the legal frameworks, professional obligations, and dominant cultural influences shaping veterinary reporting practices in South Korea, Canada, and the United States.

These observed patterns can be further understood through established frameworks in cross-cultural moral reasoning. Hofstede’s cultural dimensions theory and Triandis’s individualism-collectivism model suggest that high power distance and collectivist orientations promote social harmony but discourage confrontation with authority figures or clients [24,25]. Within such contexts, veterinarians may perceive reporting as a social transgression rather than a professional duty, thereby raising the threshold for formal action. Rest’s model of moral reasoning further explains how moral judgment schemas interact with cultural norms to shape ethical decision-making [26]. Empirical studies support this interpretation: surveys of veterinarians in Australia, New Zealand, and the United States demonstrate that cultural expectations, professional training, and perceived institutional support strongly influence the likelihood of reporting animal abuse [27,28,29,30]. In South Korea, broader cultural trends, including growing individual-level engagement with companion animal welfare and shifting public policy emphasis on welfare-oriented values, indicate a gradual move towards greater welfare awareness. Although specific data on younger veterinarians remain limited, these societal shifts may, over time, help reduce cultural barriers to formal abuse reporting.

### 5.2. Professional Autonomy and Ethical Frameworks

The association between professional autonomy and reporting obligations differs fundamentally across voluntary and mandatory systems. In South Korea’s voluntary system, veterinarians maintain complete discretion over reporting decisions, enabling tailored ethical judgments based on specific circumstances, client relationships, and cultural considerations [7]. This autonomy, while upholding professional discretion, contributes to significant underreporting when individual judgment conflicts with broader animal welfare interests.

Veterinarians from Canada and the United States operating within mandatory systems encounter different autonomy considerations, where legal obligations limit discretionary decision-making while providing clear guidelines for professional behavior. By establishing clear expectations, the mandatory framework mitigates individual ethical burden; however, it also fosters tensions between professional judgment and legal compliance. Research suggests that in mandatory reporting jurisdictions, veterinarians generally express greater confidence in their reporting decisions and experience less ethical distress when confronting suspected cases of abuse [2].

Professional ethical frameworks significantly differ across jurisdictions. Veterinary ethical frameworks in Korea customarily highlight harmonious client relationships, professional discretion, and careful consideration of social consequences, demonstrating broader cultural values [8]. In contrast, veterinary ethical frameworks in Canada and the United States prioritize animal welfare as a fundamental professional obligation, balancing client confidentiality with broader societal responsibilities and legal requirements. Professional codes of ethics explicitly state that veterinarians are duty-bound to report suspected animal abuse when legal requirements exist, placing animal welfare as a primary consideration that supersedes individual client relationships in abuse situations [2].

### 5.3. Enforcement Mechanisms and Professional Consequences

The enforcement mechanisms across jurisdictions significantly vary in terms of approach and effectiveness. South Korea’s voluntary system lacks professional consequences for nonreporting, entirely relying on individual moral conviction and professional initiative. No cases of Korean veterinarians facing disciplinary actions specifically for failing to report suspected animal abuse have been documented, indicating the absence of enforcement mechanisms within the voluntary framework [6].

In Canada, provinces implement mandatory reporting through professional licensing requirements, with veterinary colleges authorized to investigate complaints and impose disciplinary measures for noncompliance. Integrating reporting obligations into professional licensing generates systematic accountability mechanisms while providing legal protections for good-faith reporting. Typically, provincial systems encompass graduated disciplinary measures, educational requirements, and rehabilitation programs for veterinarians who fail to comply with reporting obligations.

The United States exhibits the most complex enforcement landscape, with approximately 24 states implementing various forms of mandatory reporting supported by administrative consequences. States, including California, Illinois, and Minnesota, specifically authorize license suspension or revocation for veterinarians who fail to report suspected abuse in circumstances required by law [5]. Veterinarians in states with clear enforcement mechanisms demonstrate significantly higher compliance rates and greater awareness of reporting obligations than those in voluntary reporting jurisdictions.

## 6. Discussion and Policy Recommendations

### 6.1. Effectiveness of Mandatory Versus Voluntary Systems

Comparative analysis demonstrates clear differences in effectiveness between voluntary and mandatory reporting systems. Jurisdictions with mandatory reporting requirements consistently exhibit higher compliance rates, better integration with animal welfare agencies, and more systematic approaches to abuse prevention and investigation [4]. However, mandatory systems also experience implementation challenges, including professional resistance, resource requirements for enforcement, and potential conflicts between legal obligations and professional judgment in complex situations. To achieve optimal outcomes, successful mandatory systems require comprehensive legal protections, professional education programs, and institutional support [2]. The effectiveness of mandatory systems may significantly rely on cultural context and implementation strategies. Jurisdictions with established animal welfare cultures and professional acceptance of reporting obligations exhibit smoother implementation and higher compliance rates than those attempting rapid transitions without adequate preparation.

While this review demonstrates clear differences in reporting compliance between mandatory and voluntary systems, a critical evidence gap exists regarding the direct causal relationship between increased reporting rates and measurable improvements in animal welfare outcomes. Empirical evidence directly linking these increased reports to reduced recidivism, improved animal welfare conditions, or long-term protective outcomes remains sparse. Several factors complicate this causal chain: (1) reporting represents only the initial step in a complex intervention process involving investigation, prosecution, and remediation; (2) animal welfare outcomes depend on enforcement capacity and judicial responses beyond veterinary reporting; (3) longitudinal studies tracking individual animals or perpetrators through the reporting-to-outcome continuum are methodologically challenging and largely absent from the literature [31].

The theoretical foundation supporting the reporting-welfare connection rests on several assumptions, that increased detection leads to intervention opportunities, legal consequences deter future abuse, and that removing animals from harmful situations improves welfare. While these assumptions are reasonable, they require empirical validation. Future research should prioritize longitudinal studies examining, the progression of reported cases through investigation and prosecution, recidivism rates among perpetrators in mandatory versus voluntary reporting jurisdictions, long-term welfare outcomes for animals removed from abusive situations, and the deterrent effect of mandatory reporting systems on abuse prevalence. Establishing such evidence is essential to move beyond theoretical claims and toward evidence-based conclusions regarding the effectiveness of mandatory reporting in enhancing animal welfare.

### 6.2. Policy Recommendations for South Korea

Comparative analysis with systems in Canada and the United States reveals that South Korea needs major legislative reform to address significant underreporting and enhance animal welfare protection. The following evidence-based recommendations, benchmarked against successful implementations in Canada and the United States, offer a comprehensive framework for transitioning from voluntary to mandatory reporting while addressing cultural and professional issues.

Contemporary developments in animal welfare legislation in Korea foster opportunities to implement comprehensive veterinary reporting reforms. Recent strengthening of penalties for animal abuse and expanded definitions of prohibited acts demonstrate governmental commitment to animal welfare improvement and provide regulatory foundations for mandatory reporting implementation. Building upon this legislative momentum, South Korea should strategically adapt established models from North America, starting with California’s sophisticated dual-tier strategy that discriminates between immediate reporting requirements for animal fighting cases and broader abuse reporting obligations.

This graduated framework, codified in California Business and Professions Codes §§4830.5–4830.7 [15], has demonstrated success in achieving professional compliance while minimizing implementation resistance, making it an ideal model for Korea’s cultural context where a gradual shift is more readily accepted than sudden policy reforms [16]. The integration of Canadian provincial models, particularly Ontario’s comprehensive Provincial Animal Welfare Services Act (2019), provides South Korea with a blueprint for incorporating mandatory reporting requirements with robust professional protections.

In Korea, implementation should establish clear legal safeguards that protect veterinarians who report cases from conventional litigation risks and the unique digital reputation threats prevalent in Korean online culture. Furthermore, systematic coordination between veterinary professionals and animal welfare investigation agencies offers a framework for institutional collaboration that could significantly improve the effectiveness of Korea’s currently fragmented approach to animal abuse intervention.

The administrative penalty frameworks successfully implemented in Kansas and Oklahoma reveal the significance of empowering professional associations with meaningful disciplinary authority to ensure reporting compliance. The experiences of these states show that effective mandatory reporting systems require graduated disciplinary measures that escalate from educational interventions for minor violations to license revocation for serious noncompliance cases. South Korea should enhance the current advisory role of the Korean Veterinary Medical Association by granting it comprehensive disciplinary powers, including the authority to impose license suspensions, mandate additional training, and ultimately revoke professional licenses for veterinarians who repeatedly fail to report cases of severe animal abuse.

### 6.3. Cultural Adaptation Strategies

Successful mandatory reporting implementation in South Korea necessitates careful attention to cultural factors. Community education programs should underscore connections between animal welfare and broader societal values, highlighting veterinarians’ roles as protectors while respecting traditional values of social harmony. The digital reputation management concerns unique to Korean culture require specific legislative attention. Laws should offer clear legal remedies for veterinarians experiencing online retaliation, including expedited removal procedures for malicious reviews and penalties for clients engaging in reputation attacks following legitimate abuse reports.

Financial incentives should support veterinarians reporting suspected abuse, including fee reimbursement for court testimony, practice insurance premium reductions, and continuing education credits. These incentives should offset potential economic consequences of reporting while recognizing professional contributions to animal welfare and public safety [7]. Implementation should focus on gradual cultural shifts instead of immediate comprehensive requirements. Professional organizations should lead the development of culturally appropriate protocols and offer peer support to veterinarians navigating reporting obligations.

### 6.4. Monitoring and Evaluation Systems

To track reporting patterns, investigation outcomes, and case dispositions, comprehensive monitoring systems should be implemented. Database systems should encompass provisions for anonymous reporting to protect veterinarians experiencing social pressure while maintaining accountability and transparency in investigation processes [4]. Regular evaluation of reporting system effectiveness should assess improvements in animal welfare outcomes, compliance rates, and professional satisfaction with reporting procedures. Longitudinal studies should explore changes in veterinary attitudes, reporting behaviors, and client relationships during implementation periods.

Despite ongoing evaluation of veterinary reporting mechanisms, it is important to acknowledge that animals experiencing cruelty are often not presented to veterinarians and therefore remain outside reporting statistics. As a result, most existing studies measure process outcomes, such as, reporting rates or professional compliance, rather than case outcomes, including successful intervention, enforcement, or sustained welfare improvement. Future research could expand monitoring frameworks to integrate enforcement data and animal-level welfare outcomes, allowing a more comprehensive assessment of the real-world impact of veterinary reporting system.

### 6.5. Potential Adverse Consequences for Animal Welfare

Potential adverse consequences of mandatory reporting requirements extend beyond veterinarian impacts to affect animal welfare directly. Research indicates that mandatory reporting may create barriers to veterinary care access, as owners fearing legal repercussions might delay or avoid seeking treatment for animals experiencing abuse or neglect [32]. Australian studies reveal that 76% of community members support mandated reporting, yet significant concerns exist about whether mandatory requirements might deter owners from bringing animals to veterinarians, particularly in cases where abuse results from economic hardship or lack of knowledge rather than intentional cruelty [32]. This ‘chilling effect’ could paradoxically reduce overall animal welfare by limiting preventive care and early intervention opportunities.

## 7. Conclusions

This comparative analysis reveals major differences in animal welfare protection effectiveness between voluntary and mandatory veterinary reporting systems. The success of mandatory reporting systems in Canada and the United States, where approximately 24 states implement professional disciplinary consequences (e.g., license revocation), demonstrates that legal obligations with appropriate institutional support can considerably enhance animal welfare outcomes while maintaining professional integrity [5].

Nine provinces in Canada, excluding Alberta, mandate veterinarians to report animal abuse and neglect to provincial enforcement agents, with most provinces operating comprehensive systems alongside legal protections [11]. The College of Veterinarians of Ontario states that “veterinarians have a legal and ethical responsibility to report animal abuse or neglect to a provincial animal welfare inspector when there are reasonable grounds to believe that an animal is being abused, subjected to undue physical or psychological hardship, privation, or neglect” [12].

Cultural factors play crucial roles in reporting system effectiveness, with Korean veterinarians experiencing unique challenges associated with conventional social hierarchies, digital reputation management, and professional relationship dynamics absent in other jurisdictions. The intersection of traditional Confucian values underscoring social harmony with contemporary online culture fosters complex barriers requiring specific legislative and policy attention [23].

Evidence-based policy recommendations for South Korea highlight graduated implementation of mandatory reporting requirements, comprehensive legal protections (e.g., online reputation safeguards), professional education programs addressing cultural sensitivities, and institutional infrastructure development supporting robust investigation and enforcement processes.

Future research should prioritize longitudinal studies investigating the effectiveness of different implementation strategies, cultural adaptation mechanisms, and long-term outcomes of mandatory reporting systems. Global collaboration could enable the exchange of best practices and development of culturally appropriate approaches to animal welfare legislation. Development of evidence-based policies that effectively protect animal welfare while respecting cultural diversity and professional autonomy constitutes the ultimate goal. The experiences of Canada and the United States offer valuable benchmarks for South Korea’s legislative reform initiatives, demonstrating that mandatory reporting systems with appropriate safeguards and support mechanisms show promise for improving animal welfare protection. However, establishing empirical evidence of direct causal links between reporting mandates and measurable welfare outcomes should be a priority for future research to validate these theoretical connections and guide evidence-based policy development.

## Figures and Tables

**Table 1:** Summary of analyzed materials by jurisdiction and document type (2010–2025).

Country	Legal Documents	Peer-Reviewed Articles	Government Reports	Profession Publications	Total
South Korea	8	9	3	1	21
Canada	8	10	4	2	24
United States	9	10	3	2	24
Total	25	29	10	5	69

**Table 2:** A simplified comparative overview of veterinary reporting frameworks and culture influences in South Korea, Canada, and the United States.

	South Korea	Canada	United States
Reporting Obligation	Voluntary	Mandatory	Mixed (24 states mandatory)
Disciplinary Action	None	Yes (provincial authority)	Yes (state-level in mandatory states)
Legal Protection	None	Good-faith immunity	State dependent
Cultural Factors	Harmony, online backlash, hierarchy	Strong animal welfare norms	Varies by state

## Data Availability

This narrative review is based on publicly available legal documents, peer-reviewed literature, government reports, and professional organization publications. All sources analyzed in this study are cited in the References section and can be accessed through their respective databases (PubMed, Google Scholar, Web of Science, Scopus, legal databases including Westlaw and LexisNexis, and jurisdiction-specific legislative archives). No new primary data were generated or analyzed during this research.
